# Opposite diastolic effects of omecamtiv mecarbil versus dobutamine and ivabradine co‐treatment in pigs with acute ischemic heart failure

**DOI:** 10.14814/phy2.13879

**Published:** 2018-10-11

**Authors:** Leif Rønning, Jens P. Bakkehaug, Lars Rødland, Anders B. Kildal, Truls Myrmel, Ole‐Jakob How

**Affiliations:** ^1^ Cardiovascular Research Group Institute of Medical Biology Faculty of Health Sciences UiT The Arctic University of Norway Tromsø Norway; ^2^ Cardiovascular Research Group Institute of Clinical Medicine Faculty of Health Sciences UiT The Arctic University of Norway Tromsø Norway; ^3^ Department of Cardiothoracic and Vascular Surgery, Heart and Lung Clinic University Hospital of North Norway Tromsø Norway

**Keywords:** Acute heart failure, cardiogenic shock, contractility, diastolic function, inotropic agents

## Abstract

Acute ischemic cardiogenic shock is associated with poor prognosis, and the impact of inotropic support on diastolic function in this context is unclear. We assessed two suggested new inotropic strategies in a clinically relevant pig model of ischemic acute heart failure (AHF): treatment with the myosin activator omecamtiv mecarbil (OM) or dobutamine and ivabradine (D+I). Left ventricular (LV) ischemia was induced in anesthetized pigs by coronary microembolization (*n* = 12). The animals then received OM (bolus 0.75 mg/kg, followed by 0.5 mg/kg per h) (*n* = 6) or D+I (5 *μ*g/kg per min + 0.29 ± 0.16 mg/kg) (*n* = 6), respectively. Ischemia reduced the stroke volume (SV), despite the increased left atrial pressure associated with impaired LV early relaxation, systolic dilatation, and LV late diastolic stiffness. Both treatments improved systolic ejection, but only D+I increased the SV from 26 ± 5 to 33 ± 5 mL. D+I enhanced LV early relaxation (Tau; from 45 ± 11 to 29 ± 4 msec) and prolonged the diastolic time (DT) from 338 ± 60 to 352 ± 40 msec. In contrast, OM prolonged Tau (42 ± 5 to 62 ± 10 msec) and shortened the DT (from 326 ± 68 to 248 ± 84 msec). Our data suggest that enhanced early relaxation by D+I improves LV pump function in postischemic acute heart failure. In contrast, OM worsened lusitropy in this model.

## Introduction

Left ventricular (LV) acute heart failure (AHF) following myocardial infarction is a serious and life‐threatening situation requiring urgent treatment (Hochman et al. 1999). Medical support with dobutamine is indicated for persistent compromised organ perfusion following revascularization (Ponikowski et al. 2016). A primary focus of this treatment is the restoration of contractility by improving systolic function and ventricular unloading. Little attention has been paid to understanding the diastolic pathophysiology in these patients. This is relevant because many patients experience tachycardia mediated by hypotensive baroreflex activation and the administration of *β*‐adrenergic drugs. However, tachycardia is only beneficial when it results in a significant increase in cardiac output (CO). Shortening of diastole requires an enhanced filling rate to maintain stroke volume (SV). During physiological tachycardia (i.e., exercise), the filling rate is increased as the ventricle pulls blood from the atrium, that is “diastolic suction”. The suction forces are generated during the preceding systole when the myocardium is compressed below the resting equilibrium shape. This compression generates diastolic restoring forces when the ventricle recoils and regains its passive shape and subsequently actively draws blood into the cavity (Opdahl et al. 2009). Suction is thought to be severely impaired during acute ischemia due to weakened contractility and an elevated end‐systolic volume (ESV). Thus, ventricular unloading during systole with inotrope treatment may benefit both diastolic and systolic myocardial function. A caveat to using classic inotropes is their inherent tachycardic effect exerted via the *β*‐1 receptor. In an ischemic heart with diastolic dysfunction, this effect may cause decompensation due to a discrepancy between the required filling time and the heart rate (HR). Thus, a treatment principle that selectively restores impaired contractility with a limited chronotropic response would be theoretically advantageous.

A selective HR‐reducing agent, ivabradine, has provided an opportunity to test a rate‐controlled treatment paradigm. Recent studies have demonstrated that the combined use of dobutamine and ivabradine (D+I) to treat AHF is well tolerated in patients (Cavusoglu et al. 2015) and energetically neutral for the heart (Bakkehaug et al. 2016). D+I has also been tested in the treatment of patients in cardiogenic shock (CS) (Gallet et al. 2014).

An alternative AHF treatment is a novel inotrope, omecamtiv mecarbil (OM). This drug has recently been evaluated in a phase 2 trial for the treatment of patients with AHF (Teerlink et al. 2016). OM is classified as a myosin activator, and it has been suggested to function independently of calcium currents by increasing the number of myosin heads interacting with actin filaments. This mechanism has been described as “more hands pulling on the rope” (Teerlink et al. 2011). OM extends the systolic ejection time (SET) and increases systolic unloading (reducing “end‐systolic” volume), thereby increasing the LV ejection fraction (EF). However, such an impact on the cardiac cycle might hamper diastolic function and result in an insufficient filling time, particularly during tachycardia.

In this study, we performed a comprehensive invasive assessment of diastolic function in a pig model of severe LV ischemia. The aim was to assess the therapeutic potential of omecamtiv mecarbil and dobutamine combined with ivabradine in ischemic acute heart failure.

## Materials and Methods

### Experimental animals

The experimental protocol was approved by the local steering committee of the National Animal Research Authority at the Faculty of Health Sciences, UiT The Arctic University of Norway. Castrated male domestic pigs (32.4 ± 2.2 kg) were adapted to the new environment for 5–7 days. The animals were fasted overnight and were provided water ad libitum prior to the experiments.

### Anesthesia and surgical preparation

The pigs were premedicated with intramuscular injections of 20 mg/kg ketamine (Pfizer AS, Norway), 1 mg/kg midazolam (B. Braun, Germany) and 1 mg atropine (Nycomed Pharma, Norway). Anesthesia was induced by inhalation of isoflurane (Abbot, USA). After endotracheal intubation, intravenous injections of 10 mg/kg pentobarbital sodium (Abbott, USA), and 0.01 mg/kg fentanyl (Hameln Pharmaceuticals, Germany) were administered. The animals were ventilated on positive‐pressure ventilation at 60% oxygen. An introducer sheath catheter was placed into the left internal jugular vein, and anesthesia was maintained throughout the experiment by continuous infusion of 4 mg/kg per h pentobarbital sodium, 0.02 mg/kg per h fentanyl, and 0.3 mg/kg per h midazolam. The circulating volume was maintained by 20 mL/kg per h continuous infusion of 0.9% NaCl supplemented with 1.25 g/L glucose. In addition, the animals received 2500 IU heparin (Leo, Denmark) and 5 mg/kg amiodarone (Sanofi‐Synthelabo, Sweden) to prevent blood clotting of catheters and cardiac arrhythmia, respectively. The urinary bladder was catheterized and drained by cystotomy. To enable analysis of the hemodynamic effects with minimal influence of the sympathetic nervous system, hexamethonium chloride (Sigma Chemical Co., USA) was administered as an intravenous bolus of 20 mg/kg.

A 7 Fr multisegment Millar MPVS Ultra pressure‐volume catheter (Millar, USA) was placed into the left ventricle via the common carotid artery. Arterial pressure was measured via a vascular catheter in the abdominal aorta. A 7 Fr balloon catheter (Sorin, Italy) was introduced into the inferior vena cava for preload reduction. After median sternotomy, the pericardium was removed, and the pulmonary truncus was dissected to enable the placement of a transit time flow probe (Medistim, Norway) for CO measurements. Three sonomicrometry crystals (Sonometrics Corporation, Canada) were placed in the myocardium to measure the long‐ and short‐axis myocardial dimensions. A 6 Fr introducer (Terumo, Belgium) was inserted through the thoracic wall into the left atrium, through which a 3.5 Fr pressure catheter (Millar, USA) was placed. A pacemaker electrode was sutured onto the right atrium. Epicardial echocardiography (Vivid I, GE, USA) was used to calibrate the crystal dimensions to the LV volumes at baseline.

### Experimental protocol

A full data set was obtained at three time points during the experiments: (1) pre‐ischemia, (2) ischemia, and (3) postischemic treatment. Data were obtained at intrinsic HR and during pacing‐induced tachycardia. The pacing protocol was performed in successive steps at 120 and 160 bpm, with at least 1 min between each step to allow for pressure stabilization. Measurements were performed at steady state and during an abrupt reduction in preload induced by vena cava occlusion (VCO) at each time point. Ventilation was halted in expiration during the recordings to minimize the effects of respirator‐induced alterations.

Following baseline recordings, LV ischemia was induced by repeated, fluoroscopic‐guided injections of 2–5 mL boluses of 50‐*μ*m polystyrene microspheres (2.4 mg/mL) (NEM‐005; NEN Life Science Products, USA) into the left main coronary artery. The injections were repeated until the left atrial pressure (LAP) approached 20 mmHg. An average of 11.9 ± 3.9 mL was administered over 53 ± 15 min to achieve this level of heart failure. The ischemic recordings were performed at 30 min after the last coronary injection. The animals were then split into two groups to receive either OM or D+I.

OM (Selleck Chemicals, USA) was formulated as a 1 mg/mL solution containing 50 mmol/L citrate in sterile water, and the pH was adjusted to 5.0 with NaOH (Bakkehaug et al. 2015). Based on a previously published dose–response protocol (Bakkehaug et al. 2015), a bolus dose of 0.75 mg/kg was administered over 10 min, followed by a continuous infusion of 0.5 mg/kg per h. This resulted in a targeted 20% increase in the SET, corresponding to a clinically relevant plasma concentration of 500–1000 ng/mL (Teerlink et al. 2011).

Dobutamine was infused at a dose of 5 *μ*g/kg per min supplemented with small boluses of ivabradine to attenuate the chronotropic effect. Five milligrams of ivabradine was administered via a slow intravenous bolus at 5‐min intervals until the target HR (postischemic HR) was achieved or until the overall dose of ivabradine reached 0.5 mg/kg (average dose 0.29 ± 0.16 mg/kg) (Vaillant et al. 2008). The final recordings were performed after a 30‐min stabilization period.

### Data analysis

LV pressure, transit time blood flow, sonomicrometric dimensions and vascular pressure signals were recorded, digitized, and analyzed using ADI LabChart Pro software (AD Instruments, New Zealand). Cardiac dimensions were measured at baseline and after the interventions from the short‐ and long‐axis sonomicrometry crystals, and the measurements were corrected by the epicardial short‐axis ultrasound data at baseline. The echocardiographic endocardial long axis (L_endo_) was calculated from the endocardial short axis (S_endo_) at a ratio of 1.37 (Rosner et al. 2009). The sonomicrometric signals were converted to LV end‐diastolic volume (EDV) and LV ESV using the ellipsoid formula V=*π*(S_endo_)^2^/6*L_endo_ (Zile et al. 1992). EF was calculated using the formula [EDV – ESV]/EDV * 100%. A transit time flow probe on the pulmonary artery provided CO measurements. SV was calculated from the flow probe data by CO/HR. SET was defined as the time between d*P*/d*t*_max_ and d*P*/d*t*_min_, and diastolic time (DT) was defined as the remaining fraction of the cardiac cycle. The isovolumetric relaxation constant (Tau) was calculated by Weiss’ method. To obtain the preload recruitable stroke work (PRSW), we performed transient VCO at spontaneous HR, whereas the end‐diastolic and end‐systolic pressure–volume relationships (EDPVR and ESPVR, respectively) were calculated as the linear slopes of the steady state measurements. The maximum atrial‐ventricular gradient (L _A‐V MAX_) is the peak pressure difference between the left atrium and left ventricle during diastole, and the atrial‐ventricular pressure–time integral (L _A‐V INTG_) is the area under the curve during diastole. The maximum myocardial systolic deformation rate (s′) was measured as the peak negative derivative of the long‐axis sonomicrometry crystal, whereas e′ was measured as the peak positive derivative during diastole.

### Statistics

Values are presented as the mean and standard deviation. To assess the impact of microembolization and treatment at intrinsic heart rate, the data were analyzed using paired Student's *t* test. To assess the effects of pacing induced tachycardia on treatment, the data were analyzed by two‐way mixed design ANOVA. *P* values <0.05 were regarded as statistically significant, and all analyses were conducted using SPSS version 23 (SPSS Inc., USA).

## Results

Global LV ischemia impaired early (Tau, P_min_, and d*P*/d*t*_min_) and late diastole (end‐diastolic pressure, EDP), resulting in diastolic dysfunction. Interestingly, both the L _A‐V MAX_ and L _A‐V INTG_ were maintained despite impaired early relaxation of the ischemic heart. Despite a twofold increase in the LV preload, the impaired systolic and diastolic function led to reductions in the SV, cardiac output, and mean arterial pressure.

**Figure 1:**
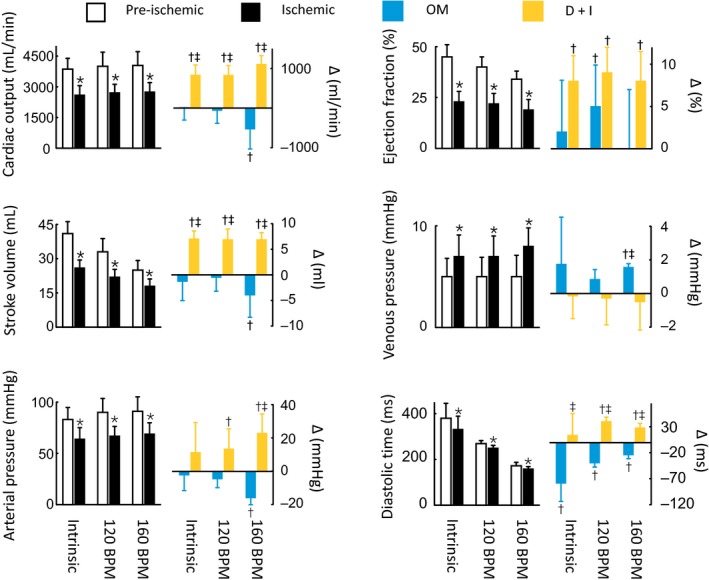
General hemodynamics at intrinsic heart rates and during right atrial pacing at 120 and 160 beats/min. The left panels show the pre‐ischemia (white bars; *n* = 12) and ischemia values (black bars; *n* = 12). The right panels show the effects of the inotropic treatments as delta changes from the ischemia values. The blue bars indicate the omecamtiv mecarbil (OM) treatment group (*n* = 6), and the yellow bars indicate the dobutamine plus ivabradine (D+I) treatment group (*n* = 6). *P < 0.05 pre‐ versus post‐ischemia (paired *t* test). ^†^ *P* < 0.05 ischemia versus treatment groups (two‐way mixed ANOVA). ^‡^ *P* < 0.05 between treatment groups (two‐way mixed ANOVA).

**Figure 2:**
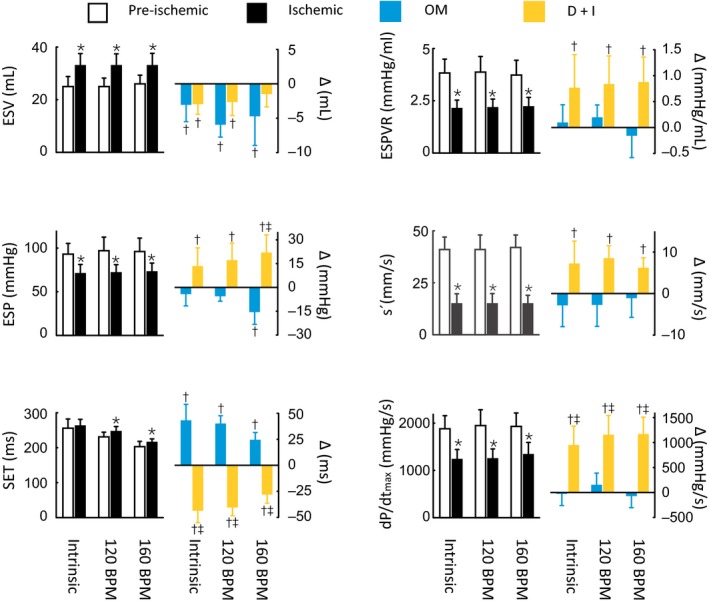
Systolic indices at intrinsic heart rates and during right atrial pacing at 120 and 160 beats/min. The left panels show the pre‐ischemia (white bars; *n* = 12) and ischemia values (black bars; *n* = 12). The right panels show the effects of the inotropic treatments as delta changes from the ischemia values. The blue bars indicate the omecamtiv mecarbil (OM) treatment group (*n* = 6), and the yellow bars indicate the dobutamine plus ivabradine (D+I) treatment group (*n* = 6). ESV, end‐systolic volume; ESP, end‐systolic pressure; SET, systolic ejection time; ESPVR, slope of steady state end‐systolic pressure–volume relationship; s′, peak LV long‐axis wall deformation velocity; and d*P*/d*t*_max_, maximum rate of LV pressure change. *P < 0.05 pre‐ versus post‐ischemia (paired *t* test). ^†^ *P* < 0.05 ischemia versus treatment groups (two‐way mixed ANOVA). ^‡^ *P* < 0.05 between treatment groups (two‐way mixed ANOVA).

**Figure 3:**
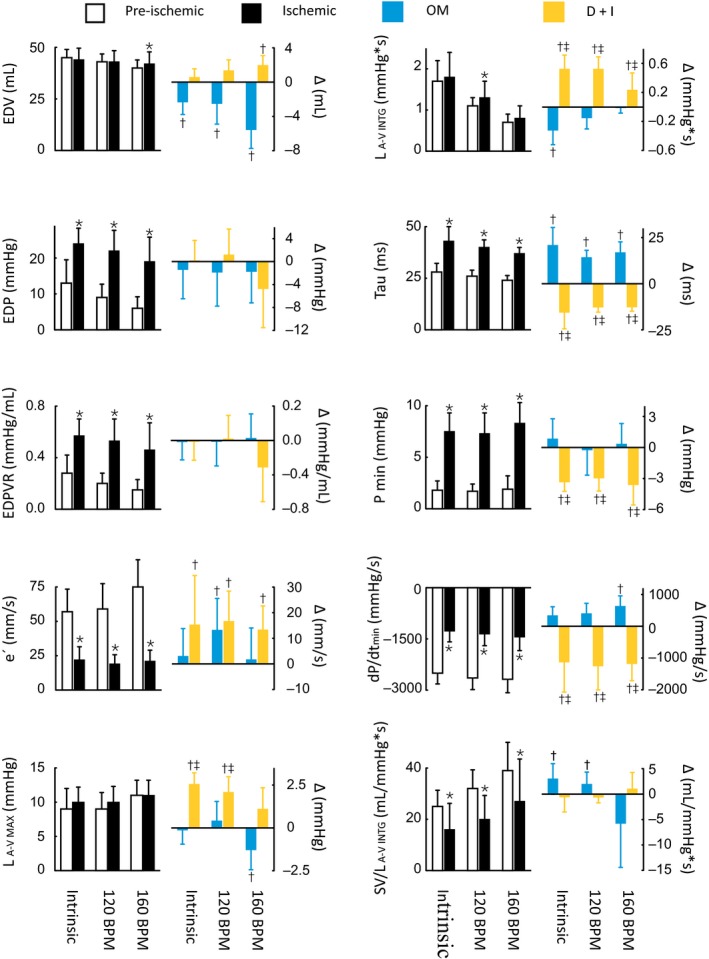
Diastolic indices at intrinsic heart rates and during right atrial pacing at 120 and 160 beats/min. The left panels show the pre‐ischemia (white bars) and ischemia values (black bars) (*n* = 12). The right panels show the effects of the inotropic treatments as delta changes from the ischemia values. The blue bars indicate the omecamtiv mecarbil (OM) treatment group (*n* = 6), and the yellow bars indicate the dobutamine plus ivabradine (D+I) treatment group (*n* = 6). EDV, end‐diastolic volume; EDP, end‐diastolic pressure; EDPVR, slope of steady state end‐diastolic pressure–volume relationship; e′, peak LV long‐axis elongation velocity; L _A‐V_ _MAX_, peak L atrial‐ventricular pressure difference; L _A‐V_ _INTG_, L atrial‐ventricular pressure–time integral during diastole; Tau, isovolumetric relaxation constant; P_min_, minimum LVP; d*P*/d*t*_min_, maximum rate of pressure decline; SV/L _A‐V_ _INTG_, SV related to the L atrial‐ventricular pressure–time integral. *P < 0.05 pre‐ versus post‐ischemia (paired *t* test). ^†^ *P* < 0.05 ischemia versus treatment groups (two‐way mixed ANOVA). ^‡^ *P* < 0.05 between treatment groups (two‐way mixed ANOVA).

Both treatments (OM and D+I) improved systolic unloading of the ischemic ventricle. The two treatments differed in three principal aspects: (1) the speed of contraction; (2) the speed of relaxation; and (3) the relationship between the systolic and diastolic times during the cardiac cycle. D+I reversed the ischemia‐induced impairments in inotropy and lusitropy, as demonstrated by a 77% increase in d*P*/d*t*_max_, an 89% increase in d*P*/d*t*_min_, and a 35% decrease in Tau (Table 1). Conversely, OM had no impact on d*P*/d*t*, and it prolonged Tau by 50%. In addition, D+I reduced the minimum LV pressure (P_min_) and thus increased the L _A‐V MAX_ during diastole. However, OM had no impact on these indices. OM biased the cardiac cycle towards systole, while D+I biased it towards diastole. The enhanced early relaxation and prolongation of diastole by D+I resulted in an increased L _A‐V INTG_ beyond the pre‐ischemia levels. This was associated by increases in the SV (27%), mean arterial pressure (18%) and ejection fraction (from 24% to 32%). In contrast, the reduction in the DT and the impaired relaxation (Tau) observed with OM administration resulted in a leftward shift of the pressure–volume loop (Fig. 5). Thus, OM failed to restore general pump indices, such as the SV, CO, mean arterial pressure, and ejection fraction. At elevated heart rate by atrial pacing the opposite effects of OM versus D+I treatment was reinforced (Figs. 1, 2, 3).

**Table 1:** Haemodynamic effects of ischemia and subsequent inotropic support from omecamtiv mecarbil (OM) or dobutamine plus ivabradine (D+I) in open‐chest pigs

	OM group (*n* = 6)			D+I group (*n* = 6)		
	Pre‐isch.	Ischemia	OM treated	Pre‐isch.	Ischemia	D+I treated
Heart rate (bpm)	100 ± 16	105 ± 16	114 ± 21	92 ± 11	99 ± 10	104 ± 8.4
Cardiac output (L/min)	3.7 ± 0.7^a^	2.6 ± 0.4	2.6 ± 0.2	4.0 ± 0.3^a^	2.6 ± 0.6	3.4 ± 0.4^a^
Stroke volume (mL)	37 ± 3.7^a^	25 ± 2.7	24 ± 3.8	44 ± 4.9^a^	26 ± 4.7	33 ± 4.8^a^
Mean arterial pressure (mmHg)	81 ± 15^a^	66 ± 10	63 ± 11	85 ± 8^a^	63 ± 15	74 ± 16
Central venous pressure (mmHg)	5.5 ± 1	6.4 ± 0.7	8.2 ± 2.9	5.7 ± 0.7^a^	8.5 ± 2.9	8.3 ± 2.0
Ejection fraction (%)	43 ± 5^a^	23 ± 6	26 ± 10	48 ± 7^a^	24 ± 5	32 ± 7^a^
Systolic ejection time (ms)	241 ± 24	239 ± 20	297 ± 29^a^	271 ± 19	272 ± 8.7	229 ± 17^a^
d*P*/d*t*_max_ (mmHg/sec)	1833 ± 297^a^	1252 ± 198	1232 ± 168	1932 ± 271^a^	1222 ± 208	2166 ± 506^a^
End‐systolic volume (mL)	26 ± 2.7^a^	36 ± 3.9	33 ± 4.5^a^	24 ± 4.7^a^	31 ± 4.9	28 ± 5.0^a^
End‐systolic pressure (mmHg)	89 ± 15^a^	72 ± 9.1	68 ± 12	97 ± 9^a^	69 ± 13	82 ± 13^a^
Left atrial pressure (mmHg)	9 ± 1^a^	22 ± 2.6	23 ± 4.1	10 ± 1.4^a^	22 ± 2.8	21 ± 3.6
d*P*/d*t*_min_ (mmHg/sec)	−2428 ± 594^a^	−1275 ± 147	−943 ± 279^a^	−2575 ± 475^a^	−1270 ± 368	−2394 ± 1037^a^
End‐diastolic volume (mL)	45 ± 7	47 ± 5.1	44 ± 5.8^a^	45 ± 3.6^a^	41 ± 5.4	41 ± 4.6
End‐diastolic pressure (mmHg)	10 ± 4^a^	23 ± 4.8	22 ± 2.5	15 ± 7.6^a^	25 ± 4.2	26 ± 4.1
Tau (Weiss) (ms)	26 ± 4^a^	42 ± 5.1	62 ± 10^a^	29 ± 4^a^	45 ± 11	29 ± 4.2^a^
Minimum pressure (mmHg)	1.6 ± 0.6^a^	8.1 ± 2.0	8.9 ± 2.4	2 ± 1.2^a^	6.9 ± 1.8	3.6 ± 1.5^a^
Diastolic time (msec)	371 ± 70^a^	326 ± 68	248 ± 84^a^	389 ± 64	338 ± 60	352 ± 40
Max atrioventricular pressure difference (mmHg)	8.4 ± 1.5	9.1 ± 2.5	8.9 ± 2.4	10 ± 1.1	10 ± 1.7	13 ± 1.3^a^
Max atrioventricular integral (mmHg × sec)	1.6 ± 0.5	1.7 ± 0.7	1.4 ± 0.6^a^	1.8 ± 0.5	2 ± 0.6	2.5 ± 0.5^a^

Data were obtained from pigs after median sternotomy with substantial cardiac instrumentation for the assessment of diastolic function. The induction of ischemia by coronary microembolization was aimed at a left atrial pressure of 20 mmHg. Following ischemic recordings, the animals were assigned to receive either omecamtiv mecarbil (0.75 mg/kg bolus + 0.5 mg/kg per h infusion) or dobutamine (5 *μ*g/kg per min) combined with ivabradine (0.29 ± 0.16 mg/kg).

a *P* < 0.05 versus ischemia (paired *t* test).

## Discussion

### Main findings

This study shows that catecholamine‐induced inotropy (D+I) and direct myosin activation (OM) had opposite effects on diastolic function in the ischemic heart. Improved diastole by D+I was attributed to enhanced early relaxation and a prolonged DT that increased the driving force across the mitral valve. In contrast, OM unloaded the heart by prolonging systole at the expense of the DT. Together with impaired early relaxation, this reduced the driving force across the mitral valve. These diastolic impairments with OM were indicated by the leftward shift of the pressure–volume loop (Fig. 5) and reduced tolerance to HR elevation (Fig. 3).

Restoring compromised SV is thought to be an important treatment target in postischemic CS. Data from the SHOCK trial (Hochman et al. 1999) indicated that stroke work and SV, which are indicators of pump function, are superior early predictors of patient survival (Jeger et al. 2007). Thus, the rationale for inotropic support as a short‐term bridge to recovery in the sickest patients may be justified despite the lack of improved outcomes in clinical trials (Ponikowski et al. 2016). Inotropes increase SV primarily by enhancing systolic unloading. The restoration of systolic function is critical because CS patients often exhibit dilated ventricles due to severely compromised contractility (Hochman et al. 1999). However, little attention has been paid to diastolic function in these patients. Using Doppler echocardiography, a sub‐study of the SHOCK trial revealed that 60.9% of the patients presented with a restrictive filling pattern (Reynolds et al. 2006), which was associated with a more severe form of shock. These patients exhibited a trend toward increased mortality, indicating that diastolic dysfunction contributed to the pathology in these patients. Other studies have also reported that patients with diastolic pathology exhibit increased risks of morbidity and mortality following acute myocardial infarction (Moller et al. 2006).

### Impact of acute ischemia on diastolic function

**Figure 4:**
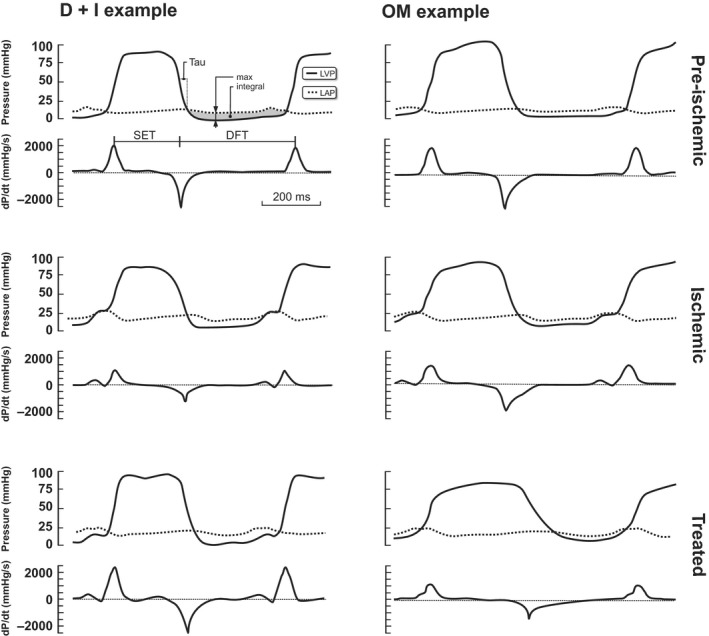
Modified Wiggers’ diagram showing examples of actual tracings from two pigs. Left column: pre‐ischemia, ischemia, and dobutamine‐ivabradine (D+I) treatment. Right column: pre‐ischemia, ischemia, and omecamtiv mecarbil (OM) treatment. LVP, left ventricular pressure; LAP, left atrial pressure; d*P*/d*t*_max_ and d*P*/d*t*_min_, maximum and minimum derivatives of the LV pressure, respectively; Tau, isovolumetric relaxation constant; P_min_, minimum LV pressure during the cardiac cycle; L _A‐V_ _MAX_, peak L atrial‐ventricular pressure difference; L _A‐V_ _INTG_, L atrial‐ventricular pressure–time integral during diastole; DT, diastolic time; SET, systolic ejection time.

Acute ischemia is known to induce diastolic dysfunction (Labovitz et al. 1987), as demonstrated by impaired and/or incomplete early relaxation or acute stiffening of the ventricle, leading to late diastolic constraint (Miyazaki et al. 1990). At the myocyte level, impaired early relaxation is related to incomplete or delayed calcium reuptake at the sarcomere, contributing to resting tension during relaxation (Westerblad and Allen 1993). Additionally, early diastolic function is dependent on restoring forces in the myocardium, which are generated by elastic recoil as the ventricle expands to regain its passive shape in early diastole (Opdahl et al. 2009). During acute ischemia, such diastolic suction is absent, as systolic unloading is incomplete (Courtois et al. 1990). Our study has confirmed this impairment in early diastole, as demonstrated by the substantial increase in the isovolumetric relaxation time, reduction in the maximum speed of relaxation and elevation in the minimum ventricular pressure during the cardiac cycle. These effects can be at least partly attributed to a lack of diastolic suction because ischemia also substantially impairs systolic unloading. Interestingly, despite the impaired relaxation rate of the LV, the peak atrial‐ventricular pressure differences were maintained in ischemia. This finding is most likely due to the twofold increase in the LAP, causing the first LAP‐LVP crossover at an earlier and steeper part of the LVP curve (see Fig. 4).

The impact of acute ischemia on ventricular distensibility in mid to late diastole appears to be multifactorial (Miyazaki et al. 1990). The underlying causes of acute stiffening in ischemia may include diastolic calcium overload (Steenbergen et al. 1987) and/or myocardial edema (Schulz‐Menger 2011). Late diastolic dysfunction was also observed in our model, as demonstrated by the substantial elevation in the EDPVR. Additionally, we assessed distensibility using a novel method. By relating the atrial‐ventricular driving force (measured as the time–pressure integral of left atrium‐ventricle during diastole) to the amount of blood entering at the same time (SV), we observed a substantial constraint against diastolic filling in the ischemic heart (Fig. 3). These findings are in line with the restrictive filling patterns often observed in severely ill CS patients (Reynolds et al. 2006).

### Effects of treatment

**Figure 5:**
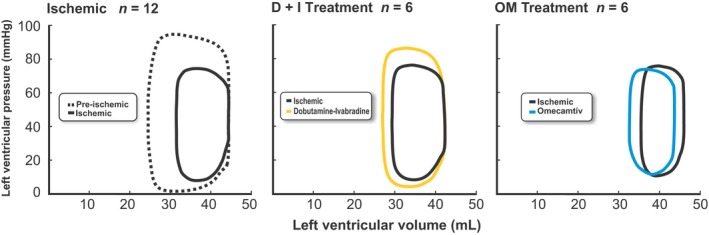
Illustration of typical pressure–volume loops at intrinsic heart rates calibrated against the mean values of ESV, EDV, EDP, P_min,_ and ESP, as shown in Table 1. The left panel shows pre‐ (dotted line) and post‐ischemia (solid line). The middle panel shows treatment with dobutamine plus ivabradine (D+I, yellow line) compared with untreated ischemia. The right panel shows treatment with omecamtiv mecarbil (OM, blue line) compared with untreated ischemia.

Ivabradine has been suggested for use as an adjunct to dobutamine in the treatment of CS (Gallet et al. 2014) to counteract undesired tachycardia. This medication does not affect inotropy, and treatment with D+I have been suggested to optimize coronary flow and reduce energy demands in the ischemic heart by optimizing the DT (De Santis et al. 2013; Gallet et al. 2014). The results obtained in the present study are in agreement with these previous findings showing that treatment with D+I increase the DT. The animals receiving either OM or D+I exhibited enhanced systolic unloading in the ischemic dilated ventricle. Therefore, both treatments should enhance restoring forces, which would facilitate suction in early diastole (Opdahl et al. 2009). However, only the pigs receiving D+I exhibited improvements in early diastolic indices (Fig. 3). In fact, with the exception of systolic unloading, the effects of the two treatments were quite opposite. This dichotomy is most likely due to the distinct effects of the drugs on the sarcomere. Catecholamines act on *β*‐adrenergic receptors to enhance transient cytosolic Ca^2+^ amplitude (inotropy) and increase the rate of sarcoplasmic reticulum Ca^2+^ reuptake (lusitropy) (Bers 2002). The intracellular ion fluxes can be mirrored in the whole heart by measuring d*P*/d*t*_min_ and d*P*/d*t*_max_. These findings were confirmed in our study, which showed that ischemia reduced the speed of LVP acceleration and deceleration, which was restored by administration of the *β*‐agonist dobutamine in combination with ivabradine. However, pro‐arrhythmogenic effects and a potentially increased energetic cost (Muller et al. 2010) are associated with the use of sympathomimetic drugs. These disadvantages have prompted the research and development of alternative inotropes, such as calcium sensitizers and myosin activators, that do not alter intracellular calcium levels. The sensitizer levosimendan has reached clinical application, but studies have indicated that its inotropic effects may be attributed to PDE3 inhibition (Ørstavik et al. 2014). At present, the myosin activator OM has not been demonstrated to modulate calcium (Malik and Morgan 2011), which is in line with the unaltered d*P*/d*t*_max_ observed in the OM‐treated animals in this study. Instead, OM treatment improves systolic unloading by prolonging the SET (Malik and Morgan 2011). In cardiomyocytes, this is evident as an abnormal inotropic response with a negative treppe (force–frequency relationship) and offset in the relationship between intracellular calcium amplitude and sarcomere shortening (Butler et al. 2015). Our data indicate that the diastolic consequence of this mechanism of action is prolonged and slowed relaxation. This is in line with the previous finding that OM slows relaxation and increases passive tension at rest in isolated cardiomyocytes (Nagy et al. 2015). In intact subjects, these effects are reflected by an increased Tau and reduced d*P*/d*t*_min_. In addition, the consequence of an increased SET is a reduced DT. These findings highlight underlying mechanisms that could explain why OM fails to restore the SV in ischemic hearts. And indeed, when DT was further shortened by tachycardia, OM treatment caused a general hemodynamic decompensation (Fig. 1). The constrain with OM treatment is also displayed by that improvements in systolic unloading are counteracted by impairments in diastolic filling, as demonstrated by the leftward shift of the entire pressure–volume loop (Fig. 5).

## Conclusion

Our data demonstrated that controlling tachycardia with ivabradine optimizes the lusitropic effect of dobutamine in ischemic acute heart failure. In contrast, inotropic support with omecamtiv mecarbil is limited by the impairment in diastolic function that is reinforced at elevated heart rates.

## Conflict of Interest

The authors have no conflicts of interest to declare.
